# Heterologous Expression of a Soybean Gene *RR34* Conferred Improved Drought Resistance of Transgenic *Arabidopsis*

**DOI:** 10.3390/plants9040494

**Published:** 2020-04-12

**Authors:** Duong Hoang Trong Nghia, Nguyen Nguyen Chuong, Xuan Lan Thi Hoang, Nguyen Cao Nguyen, Nguyen Huu Cam Tu, Nguyen Van Gia Huy, Bui Thi Thanh Ha, Thai Nguyen Hoang Nam, Nguyen Binh Anh Thu, Lam-Son Phan Tran, Nguyen Phuong Thao

**Affiliations:** 1 Applied Biotechnology for Crop Development Research Unit, School of Biotechnology, International University, Ho Chi Minh City 700000, Vietnam; dhtnghia@gmail.com (D.H.T.N.); nguyenchuong1402@gmail.com (N.N.C.); htlxuan@hcmiu.edu.vn (X.L.T.H.); ncn0809@gmail.com (N.C.N.); mikanguyen1201@gmail.com (N.H.C.T.); nvghuy.esc@gmail.com (N.V.G.H.); thanhha271193@gmail.com (B.T.T.H.); namhoang909@gmail.com (T.N.H.N.); nbathu192@gmail.com (N.B.A.T.); 2 Vietnam National University, Ho Chi Minh City 700000, Vietnam; 3 Institute of Research and Development, Duy Tan University, 03 Quang Trung, Da Nang 550000, Vietnam; son.tran@riken.jp; 4 Stress Adaptation Research Unit, RIKEN Center for Sustainable Resource Science, 1-7-22, Suehiro-cho, Tsurumi, Yokohama 230-0045, Japan

**Keywords:** drought, *GmRR34*, response regulator, soybean, two-component system

## Abstract

Two-component systems (TCSs) have been identified as participants in mediating plant response to water deficit. Nevertheless, insights of their contribution to plant drought responses and associated regulatory mechanisms remain limited. Herein, a soybean response regulator (RR) gene *RR34*, which is the potential drought-responsive downstream member of a TCS, was ectopically expressed in the model plant *Arabidopsis* for the analysis of its biological roles in drought stress response. Results from the survival test revealed outstanding recovery ratios of 52%–53% in the examined transgenic lines compared with 28% of the wild-type plants. Additionally, remarkedly lower water loss rates in detached leaves as well as enhanced antioxidant enzyme activities of catalase and superoxide dismutase were observed in the transgenic group. Further transcriptional analysis of a subset of drought-responsive genes demonstrated higher expression in *GmRR34*-transgenic plants upon exposure to drought, including abscisic acid (ABA)-related genes *NCED3*, *OST1*, *ABI5*, and *RAB18*. These ectopic expression lines also displayed hypersensitivity to ABA treatment at germination and post-germination stages. Collectively, these findings indicated the ABA-associated mode of action of GmRR34 in conferring better plant performance under the adverse drought conditions.

## 1. Introduction

Being immobile, plant growth and development are vulnerable to environmental changes [1]. Under overpopulation and climate change pressures, water crisis has become the major concern of many countries around the world and affects multiple aspects of human life including health care, environment and food availability [2,3,4]. In agriculture, various measures have been engaged to minimized yield loss due to limited water supply, including modification of irrigation methods and employment of biotechnology [3,5,6]. In recent decades, genetic engineering has appeared as a practical strategy to deal with water scarcity, upon which novel cultivars with better drought tolerance are developed based on the comprehensive understanding of plant responses under stress conditions and identification of key genes for genetic manipulation [7,8,9,10].

Plants have adopted a variety of defense mechanisms that ultimately lessen the negative effects of environmental adversities on plant growth and productivity [11]. These activities are involved in various molecular, biochemical, and physiological adjustments [12], and are under regulation of various stress-related signaling pathways [13,14,15,16]. Although the participants, roles and mechanisms might be differed, a general scheme is shared among these signaling pathways, which are involved in the perception of external stimuli, transduction of signal molecules and regulation of gene expression [17,18,19]. Furthermore, a number of overlapping components and crosstalk among different pathways have been identified, suggestively to form a dynamic network in synchronically mediating plant responses to the tough adverse conditions [16,19,20]. 

Two-component system (TCS) is one of the most evolutionarily conserved signaling cascades present in various living organisms, from prokaryotes to eukaryotes [21]. Due to its contributions in many different biological processes, TCS studies have attracted much attention. For example, TCSs have been found to be involved in megagametogenesis, light perception, flowering, osmosensing as well as in ethylene- and cytokinin (CK)-signaling pathways [14,22,23,24,25]. Additionally, genome-wide studies of TCSs have been carried out in a variety of plant species, including chickpea (*Cicer arietinum*) [26], tomato (*Solanum lycopersicum*) [27], cucumber (*Cucumis sativus*) [28], banana (*Musa acuminata* and *Musa balbisiana*) [29], and soybean (*Glycine max*) [30]. A canonical TCS comprises of two basic components, which are a histidine kinase (HK) as receptor of signal input and a response regulator (RR) as mediator for a response output [21,23]. A third component, namely histidine-containing phosphotransmitter (HP), is seen in more complex TCSs, whereby it can be a part of HK (so-called hybrid HK) or as a separate component [22]. Signal transduction via TCS is achieved by transferring the phosphoryl group from His residue of CHASE (cyclase/HK-associated sensory extracellular) domain of the HK to the HP and subsequently to Asp residue of the RR, resulting in a corresponding response [21,23,30].

Under abiotic stress such as drought condition, TCS members have been found to participate in regulating plant responses [31]. In *Arabidopsis*, AHK1, a CK-independent HK, was indicated as a positive regulator of drought [32,33] whereas three CK-dependent HKs (AHK2, 3 and 4) were found to negatively regulate the drought responses [31]. Loss-of-function study of *Arabidopsis* type-B RRs (*arr1,10,12*) also demonstrated the negative roles of ARR1, 10 and 12 in plant response to drought [34]. Previously, Tran and colleagues had identified several members of the *TCS* family in soybean which may play a role in soybean response to water-deficit conditions [35]. Among these genes, *GmRR34*, a type-C *RR*, was found to be intensively induced upon exposure to 10-h-dehydration treatment. A subsequent study of *TCS* genes using Vietnamese drought-tolerant and drought-sensitive soybean cultivars revealed nine genes (i.e., 2 HK-, 1 HP-, and 6 RR-encoding genes) that might have positive involvement in mediating plant adaptation to water-deficit conditions, as their expression levels were enhanced significantly in the former cultivar [36]. Interestingly, *GmRR34* was also a member of this candidate list. Among the four different classes of RRs in plants, type-A and B RRs have been intensively studied as they are the two largest groups involved in CK signaling pathway [14,20]. On the other hand, little is known about the remaining groups of RRs, which include type-C RRs. Therefore, in this study, we analyzed phenotypic, physiological, biochemical, and molecular traits of transgenic *Arabidopsis* plants ectopically expressing *GmRR34* to clarify its role and mechanism in mediating plant response to drought.

## 2. Results

### 2.1. Ectopic Expression of GmRR34 Altered the Growth Characters in Transgenic Arabidopsis Plants

**Figure 1:**
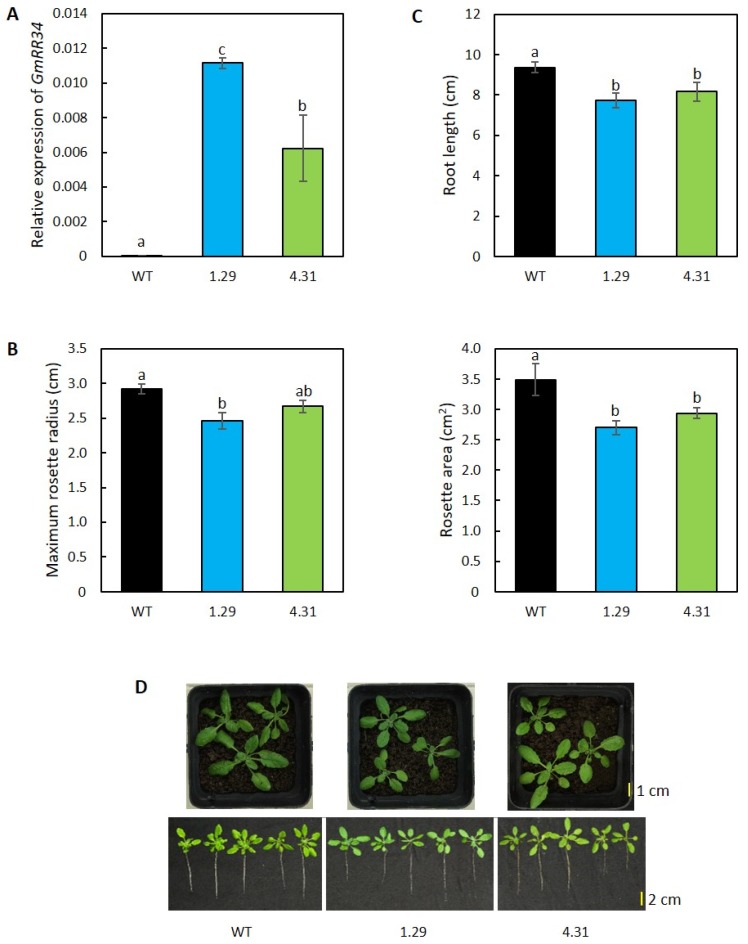
Transgene expression and phenotypic characteristics of *Arabidopsis* ectopically expressing *GmRR34* (independent lines 1.29 and 4.31) under normal growth conditions. (**A**) Relative expression level of transgene in the ectopic expression lines using *GmRR34*-specific primers. Wild-type plants were used as the control sample (*n* = 3). (**B**) Maximum rosette radius and average rosette area (*n* = 10). (**C**) Root length (*n* = 10). (**D**) Overall rosette and primary root phenotypes. Error bars indicate standard errors. Statistically identified differences among three genotypes under the same treatment were indicated by different letters (*p*-value < 0.05).

In this study, we used heterologous expression system to characterize the biological functions of GmRR34 associating with plant tolerance to water deficit. The screening for successfully transformed homozygous transgenic plants identified two independent lines (1.29 and 4.31). These transgenic plants were then subjected to the examination of *GmRR34* expression and phenotypic characteristics. Relative expression of *GmRR34* under normal growth condition, measured by RT-qPCR, confirmed the transgene expression in the transgenic lines although a non-specific product was also detected in the WT plants at very low expression level (Figure 1A). It is observed that the amount of *GmRR34* transcripts in line 1.29 was nearly double of that in line 4.31. Under normal conditions, both ectopic expression lines exhibited a reduced growth phenotype when compared with the wild-type (WT) plants. According to the analysis, the transgenic group had smaller average rosette radius and area by approximately 16% and 23%, respectively, in line 1.29, and by around 10% and 16%, respectively, in line 4.31 than did the WT (Figure 1B,D). Consistently, shorter root lengths by ca. 17% in 1.29 and 13% in 4.31 were also observed in comparison with the WT (Figure 1C,D).

### 2.2. Ectopic Expression of GmRR34 Improved Post-Drought Survival Rates in Transgenic Arabidopsis

**Figure 2:**
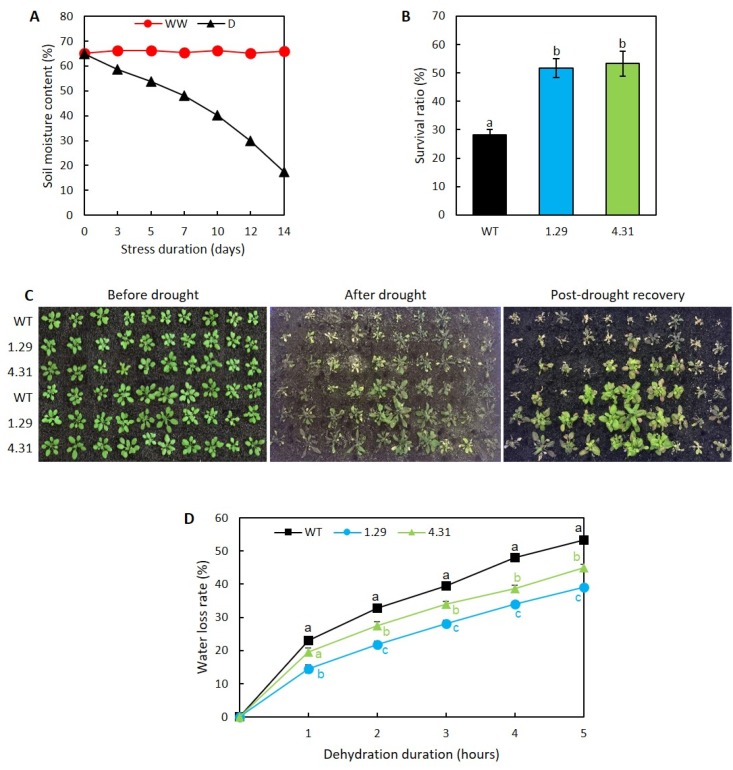
Survival test and water loss assay of transgenic *Arabidopsis* ectopically expressing *GmRR34* (independent lines 1.29 and 4.31). (**A**) Monitored soil moisture content (SMC) during drought treatment for survival test (*n* = 6 reading points). (**B**) Post-drought survival ratios of wild-type (WT) and transgenic plants recorded after re-watering for 3 days (*n* = 3 replicates per genotype, 20 plants per replicate). (**C**) Phenotype of WT and transgenic plants at different stages of survival test. (**D**) Average water loss rates from detached aerial parts from 24-day-old plants and left air-dried for 5 h (*n* = 9). Error bars indicate standard errors. Statistically identified differences among three genotypes under the same treatment were indicated by different letters (*p*-value < 0.05). WW, well-watered and D, 14-day-drought-treated conditions.

To evaluate the drought resistance mediated by the ectopic expression of *GmRR34*, drought treatment was applied to evaluate survival ratio of each genotype. After 14 days of water withholding (i.e., soil moisture content (SMC) dropped from 65% to 17%, Figure 2A), both transgenic lines demonstrated their remarkable drought resistance when compared with the WT’s capacity. After 3 days of re-watering, the survival ratios were 52% and 53% for ectopic overexpression lines 1.29 and 4.31, respectively, which were significantly higher than that of the WT (merely 28% survived) (Figure 2B,C). Additionally, examination of water loss rates from detached aerial tissues of transgenic *Arabidopsis* versus WT plants was conducted. As shown in Figure 2D, the results further illustrated the *GmRR34*-transgenic plants with better capability of retaining water over the course of dehydration treatment. According to our findings, the degrees of fresh weight (FW) reduction observed in both lines 1.29 and 4.31 were significantly lower than in WT at the same examined time points (e.g., water loss rate of 53% in WT compared with 39% in 1.29 and 45% in 4.31 upon 5-h-dehydration treatment). These results showed that line 1.29 displayed the lowest water loss rate (Figure 2D). 

### 2.3. GmRR34-Ectopic Expression Lines Displayed More Active Enzymatic ROS-Scavenging Activities

**Figure 3:**
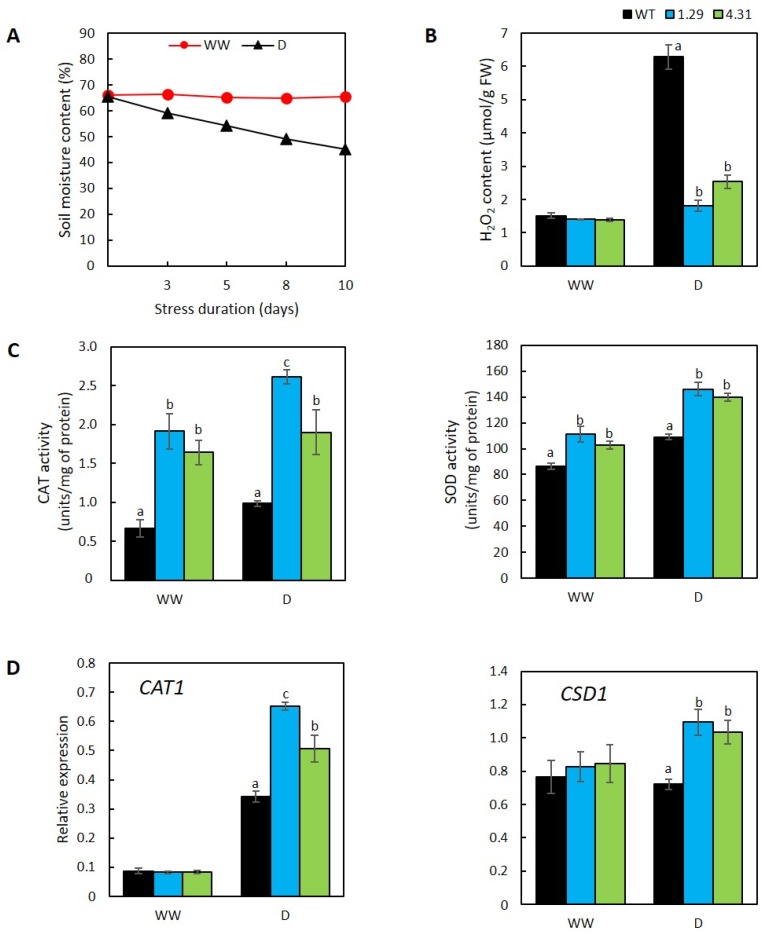
Evaluation of catalase (CAT) and superoxide dismutase (SOD) enzyme activities in wild-type (WT) and transgenic *Arabidopsis* ectopically expressing *GmRR34* (independent lines 1.29 and 4.31). (**A**) Monitored soil moisture content (SMC) during the stress assay (*n* = 6 reading points). (**B**) Endogenous hydrogen peroxide (H_2_O_2_) contents (*n* = 3). (**C**) Activities of CAT and SOD enzymes (*n* = 3). (**D**) Relative expression levels of *AtCAT1* and *AtCSD1* (*n* = 3). Error bars indicate standard errors. Statistically identified differences among three genotypes under the same treatment were indicated by different letters (*p*-value < 0.05). WW, well-watered; D, 10-day-drought conditions.

Endogenous level of hydrogen peroxide (H_2_O_2_) in WT and transgenic *Arabidopsis* was investigated to evaluate the degree of cellular damage caused by drought effects. According to our study, the WT plants exposed to drought displayed 3.5- and 2.5-fold higher levels of endogenous H_2_O_2_ than the corresponding levels measured in transgenic lines 1.29 and 4.31, respectively (Figure 3A,B). As antioxidative enzymes are known to be involved in the reactive oxygen species (ROS) detoxification process [15], we further examined the common ROS-scavenging enzymes catalases (CAT) and superoxide dismutase (SOD). The biochemical analyses demonstrated that the transgenic plants were better at detoxifying ROS than WT, since both lines 1.29 and 4.31 showed enhanced CAT and SOD activities, not only under drought but also well-watered conditions, in comparison with the corresponding enzyme activities in the WT (Figure 3C). Another notice was the more pronounced degree of SOD induction upon the stress application (increased by 31% in line 1.29 and 35% in line 4.31 versus 26% in WT). Additionally, our RT-qPCR results revealed the enhanced expression of genes coding for these enzymes. Relative expression levels of CAT- and SOD- encoding genes, *CAT1* (catalase1) and *CSD1* (copper/zinc superoxide dismutase 1) [37,38], were found to be substantially higher in the transgenic *Arabidopsis* when compared with the corresponding levels in the WT plants under the drought treatment (Figure 3D). Specifically, *CAT1* expression was up-regulated by drought by 7.9-fold in line 1.29 and 6.1-fold in line 4.31 versus 3.9-fold in WT, whereas the induction levels in expression of *CSD1* upon the stress challenge were 1.3-fold (line 1.29) and 1.2-fold (line 4.31) compared with 0.9-fold in WT.

### 2.4. Stress-Related Genes with Enhanced Expression in GmRR34-Transgenic Arabidopsis

**Figure 4:**
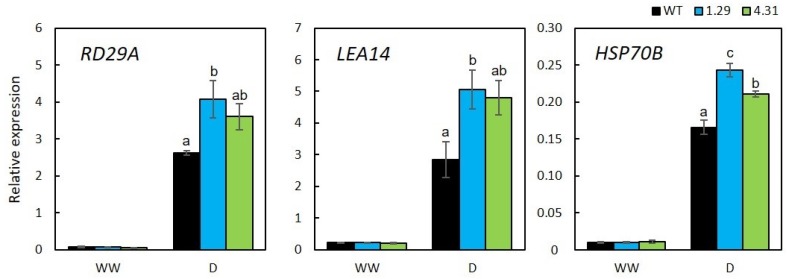
Relative expression of drought-responsive genes *AtRD29A*, *AtLEA14* and *AtHSP70B* under well-watered (WW) and 10-day-drought (D) conditions in wild-type (WT) and transgenic plants ectopically expressing *GmRR34* (independent lines 1.29 and 4.31). Three biological replicates were used for each genotype under the same treatment, and statistically identified differences among them were indicated by different letters (*p*-value < 0.05). Error bars indicate standard errors.

To obtain further molecular insights regarding GmRR34 function in mediating plant resistance to drought, we also examined the expression of three well-known drought-responsive marker genes by RT-qPCR, including *RD29A* (responsive to desiccation 29A), *LEA14* (late embryogenesis abundant 14) and *HSP70B* (heat shock protein 70B). Statistical analyses of the expression of these genes showed a significantly higher accumulation of their transcripts when compared with those in the WT counterparts during water deficit, albeit no differences in the relative expression could be observed under normal conditions. Among these genes, *RD29A* exhibited the highest up-regulation level upon drought treatment (i.e., 51- and 68-fold induction in 1.29 and 4.31 lines, respectively) while the other two genes *LEA14* and *HSP70B* showed similar increase in expression profile, which was around 20-fold in both transgenic lines (Figure 4).

### 2.5. GmRR34 Ectopic Expression Lines Displayed Hypersensitivity to ABA 

**Figure 5:**
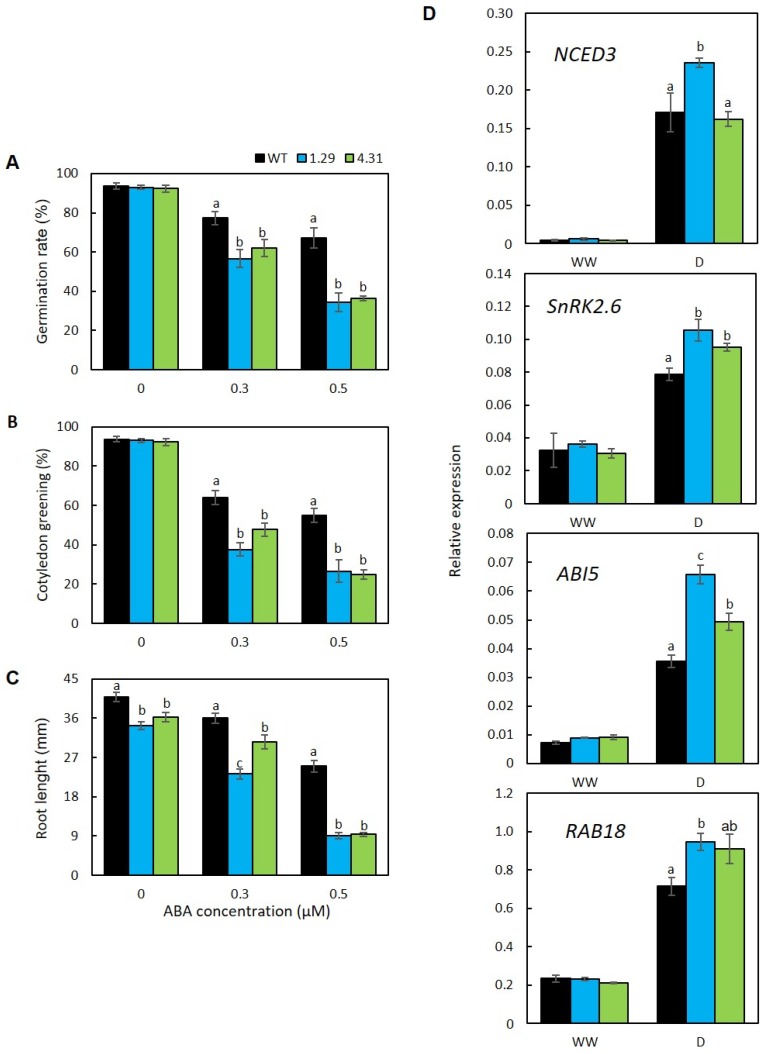
ABA sensitivity assays and expression of ABA-related genes in wild-type (WT) and transgenic plants ectopically expressing *GmRR34* (independent lines 1.29 and 4.31). (**A**) Germination rate, (**B**) green cotyledon rates (*n* = 3 replicates per genotype per treatment, 100 seeds per replicate) and (**C**) root length (*n* = 10) of WT and transgenic plants on MS medium supplied with different concentrations of ABA (0, 0.3 and 0.5 µM). (**D**) Relative expression levels of *AtNCED3*, *AtSnRK2.6*, *AtABI5* and *AtRAB18* under well-watered (WW) and 10-day drought (D) conditions (*n* = 3). Error bars indicate standard errors. Statistically identified differences among three genotypes under the same treatment were indicated by different letters (*p*-value < 0.05).

Sensitivities of the transgenic plants to abscisic acid (ABA) were examined by various parameters, including evaluation of germination rate, cotyledon development and root elongation of young seedlings upon exogenous ABA application. According to our results, all examined genotypes grown on MS medium without ABA supplementation shared similar rates of germination (approximately 93%). Nevertheless, the two transgenic lines exhibited highly repressed germination rates in comparison with that of WT upon the application of the same ABA concentrations (Figure 5A). With 0.3 µM of ABA, germination rates of the ectopic expression lines were around 20%–27% lower than that of WT. Under higher exogenous ABA condition (0.5 µM), the transgenic group showed substantial suppression to germination, whereby their germination rates were approximately a half of that of the WT. The recorded proportions of cotyledon greening also consistently illustrated hypersensitivity of *GmRR34*-transgenic plants to ABA (Figure 5B). Although comparable percentages of seedlings with greening cotyledon at roughly 93% were observed among the genotypes under the control conditions, there was a decline in number of seedlings with successful cotyledon development upon ABA treatments, and with higher proportions in the transgenic lines in comparison with that of the WT. As shown in Figure 5B, the percentages of plants with cotyledon greening were decreased by 2.5- and 3.5-fold in line 1.29, and 1.9- and 3.7-fold in line 4.31 in the media supplemented with 0.3 and 0.5 µM ABA, respectively. In agreement with these data, inhibition of root elongation in the presence of ABA was more severe in the transgenic group (i.e., reduced by 16%–32% to 72%–73% in the transgenic lines versus 11% to 39% in WT at 0.3 and 0.5 µM ABA, respectively) (Figure 5C and Supplementary Figure S1). Additionally, the expression of several key genes belonging to ABA biosynthesis and signaling pathway, including *NCED3* (nine-cis-epoxycarotenoid dioxygenase 3), *OST1*/*SnRK2.6* (open stomata 1/snf1-related protein kinase 2.6), *ABI5* (ABA insensitive 5) and *RAB18* (responsive to ABA 18), was evaluated by RT-qPCR. The data showed that expression of these genes was enhanced in the transgenic lines. Under the drought conditions, apart from *NCED3* for which solely the 1.29 ectopic expression line exhibited significantly higher expression level than did the WT, the expression levels of other examined ABA-responsive genes in both overexpression lines were remarkably higher by an average of 40% in comparison with corresponding levels in WT plants experiencing the same drought treatment (Figure 5D).

## 3. Discussion

Previous studies have provided strong lines of evidence for the participation of the soybean type-C *RR* gene *GmRR34* in drought stress responses [35,36]. According to these reports, expression of *GmRR34* was markedly up-regulated in root and/or shoot tissues under drought [36] and dehydration [35]. Furthermore, sequence analysis of *GmRR34* recognized a *cis*-motif MYCR (myelocytomatosis-related protein recognition site) in its 1000-bp promoter region [30]. MYC transcription factor (TF) family has been known to act in drought-responsive pathway in an ABA-dependent manner [17,39]. These findings suggested that *GmRR34* might contribute a major role in soybean response to water deficit, thus conferring the plant resistance to drought stress. 

In this study, we aimed to explore the biological functions of GmRR34 in relation to drought responses by using the heterologous system. The expression of *GmRR34* in the two homozygous independent transgenic *Arabidopsis* lines that we successfully screened out was confirmed by RT-qPCR (Figure 1A). The findings showed that the expression levels of *GmRR34* in lines 1.29 and 4.31 were 809- and 452-fold higher, respectively, than the expression level detected in WT plants, when using the *GmRR34* primers. Previous bioinformatic analyses revealed that a *GmRR34* homolog, *AtRR24*, was found in *Arabidopsis* genome [30]. This helps explain why a non-specific product has been amplified in the WT plants. In addition, our findings indicated that the ectopic expression of *GmRR34* resulted in retarded growth in the transgenic plants under normal growth conditions (Figure 1B–D). Previous studies also reported on similar phenotypic alteration in relation to improved drought tolerance/resistance when using constitutive *35S* promoter to drive the expression of transgenes in various transgenic species, including *Arabidopsis* [40,41,42], tobacco [43,44], and tomato [45,46].

Results from survival test illustrated that the transgenic plants ectopically expressing *GmRR34* acquired better performance against 14-day-drought treatment than their WT counterpart, as the number of transgenic plants in each genotype that could continue their life after drought treatment and re-watering was nearly double that of the WT (Figure 2B,C). The transgenic lines displayed lower water loss rates from detached aerial parts than their WT counterpart, which could be associated with their smaller rosette phenotype (Figure 1B–D and Figure 2D). In plant adaptation to drought, inhibition in shoot and leaf growth and thus reduced transpiration was promoted due to alterations in concentrations of various phytohormones, including ABA, CK and gibberellin [47,48,49,50,51]. Therefore, better cellular water conservation is an important attribute to plant survival under water-deficit conditions [41,52]. Examining water loss rate from detached leaves/rosettes has been widely used as important, reliable indicator to rapidly predict the plant water status and performance under drought conditions [53,54,55]. A great number previous studies included this tool in evaluating the performance of various transgenic plants, with the tracking of water loss rates over the course duration of 3 h [56,57], 4 h [58,59], 5 h and longer [52,60]. Upon being excised, water loss is indicated to be achieved via stomatal transpiration within the first hour followed by cuticular transpiration and residual transpiration (i.e., from incompletely closed stomata) in subsequent hours [61]. From our data, in the first hour, WT transpired more than transgenic plants, and line 1.29, with higher *GmRR34* expression, transpired the lowest. This could indicate a direct or indirect relationship with stomatal conductance regulation. Additionally, differences after the first hour tend to remain, indicating a decreased cuticular transpiration in the transgenic plants (Figure 2D). It is also worth noting that depending on duration and intensity of a certain drought condition, different responsive mechanisms might be used by the plants.

Drought stress often induces oxidative stress due to a burst production of ROS, which are the causes for cellular structural damage and biological function impediment [62,63]. Despite being a toxic by-product of cellular metabolism activities, under normal growth conditions, ROS levels are delicately maintained at a steady and low concentrations by ROS-scavenging activities as they contribute major parts in various signaling pathways [15,64,65]. During drought events, in addition to the electron transport chain activities in the chloroplast, photorespiration which is caused by stomatal closure is another source for extensive ROS production, especially H_2_O_2_ [66,67]. Therefore, endogenous H_2_O_2_ levels and detoxification activities by enzymes are commonly measured as important parameters in drought tolerance studies [40,42]. As 14-day-drought duration used in the survival test would be too severe and thus cause difficulties in analyzing biochemical data due to protein degradation and plant death, we determined to use a shorter stress duration (i.e., 10 days) for these assays. In agreement with higher survival ratios and lower water loss rates, both *GmRR34*-transgenic lines appeared to be “less stressed” with lower endogenous H_2_O_2_ contents and higher activities of CAT and SOD enzymes (Figure 3B,C). Furthermore, expression analyses of genes encoding CAT and SOD revealed significantly higher transcript abundance in the ectopic expression lines (Figure 3D). These data indicate that the lower H_2_O_2_ contents in lines 1.29 and 4.31 versus the WT could be the result of higher CAT activities and *CAT1* expression in the former group [68]. *CAT1* and *CSD1* are known to encode ROS-scavenging enzymes CAT and SOD, respectively, which catalyze the conversion of H_2_O_2_ and superoxide (i.e., another type of ROS), accordingly [37,38]. These results altogether imply that the transgenic plants were better protected from damages by ROS accumulation. As there might be other gene members of *CAT* and *SOD* families which are also involved in mediating plant response to drought [37,69], it is not surprised when the biochemical and expression data were not entirely correlated. In *Arabidopsis*, three CAT members (CAT1, CAT2, and CAT3) have been reported to participate in decomposition of H_2_O_2_ and regulating ROS homeostasis [70]. While *CAT1* expression has been linked with various stress conditions such as drought, in normal growth, the majority of CAT activities in controlling ROS homeostasis are the results of *CAT2* and *CAT3* [37,70]. Similarly, detoxification of superoxide was found to be the result of seven SOD enzymes, three of which were copper/zinc superoxide dismutases (CSD1, CSD2, and CSD3) [69]. Therefore, investigation on expression of other CAT- and SOD-encoding gene members under drought context should be conducted in future studies.

Three well-known stress-responsive genes, *RD29A*, *LEA14*, and *HSP70B*, were also selected to examine their expression in our study to gain insights to the molecular function basis of *GmRR34*. Expression patterns of these genes have been considered important indicators for drought-responsive studies [71,72]. As shown in Figure 4, under drought condition, expression levels of all these genes were significantly higher in the transgenic lines compared with those in WT by 30% (*HSP70B*) to nearly 50% (*RD29A* and *LEA14*). Studies have reported rapid induction of the stress marker gene *RD29A* under various abiotic stresses, such as water deficit, and particularly in stress-tolerant plants, thus emphasized its role as indicator for resistance toward drought [71,73,74]. Herein, in correlation with higher resistance to drought of transgenic plants (Figure 2), *RD29A* expression was also found to be remarkedly induced, suggesting *RD29A* is one of GmRR34 target for mediating drought responses, through a direct and/or indirect manner. LEA14 and RAB18 belong to LEA family, a group of proteins that play important roles in protecting cellular proteins and membrane stabilization under osmotic stress conditions [72,75]. On the other hand, HSP70B, of which expression was found to be highly expressed under extreme temperature conditions [76], belongs to a major HSP family and acts as a molecular chaperone and folding catalyst [77]. *HSP70B* has been previously demonstrated to be regulated by drought-related TFs, including MYB21 [78]. The fact that *HSP70B* was recorded to be noticeably induced upon drought in our study suggests its conserved roles in maintaining protein structures not only under heat stress but also under water stress conditions. Comprehensively, higher induction of the studied stress-responsive genes in transgenic plants ectopically expressing *GmRR34* implied the beneficial role of GmRR34 in plant resistance to drought.

According to our findings, both lines of transgenic *Arabidopsis* were hypersensitive to the presence of exogenous ABA, illustrated by substantially lower rates of germination and seedlings with normal cotyledon development as well as reduced root growth (Figure 5A–C). Ever since its discovery, ABA has been intensively studied and its functions have been linked to various stress responses, from water relation such as drought, cold or heat to wounding or pathogen infection, by inducing stomatal closure or regulating the expression of ABA-responsive genes [17,79,80,81]. Thus, such ABA-hypersensitive traits imply ABA-dependent activities of GmRR34 and possibly faster responses to osmotic changes in the transgenic plants. Known ABA-mediated stress regulations include guard cell responses or production of osmo-compatible solutes, which play major roles in retaining water from evaporation and maintaining cellular water potential [72,79,82]. 

Similar to findings for the expression pattern of *RD29A*, *LEA14*, and *HSP70B*, expression levels of ABA-related genes were comparable under normal condition among all genotypes but at substantially higher levels in the transgenic plants under drought (Figure 4 and Figure 5D). This observation could be explained that the constitutive expression of *GmRR34* under non-stressed condition was not sufficient to trigger alteration in expression of these studied genes, whereas upon the stress challenge, activities of these genes could be under the control of a complicated multi-component mechanism related to drought response, suggestively including GmRR34, that are worthy to be elaborated more in the forthcoming studies. The higher expression of ABA-related genes in the ectopic expression lines implies certain advantages acquired by the plants under drought conditions. *NCED3*, which codes for 9-*cis*-epoxycarotenoid dioxygenase, is a key enzyme for ABA biosynthesis [83]. Among the seven cloned *AtNCED*s in *Arabidopsis*, *NCED3* was the only gene induced by drought [83]. From our results, the fact that expression of *NCED3* was highly induced along with the outstanding expression of *GmRR34* in transgenic line 1.29 under drought conditions suggests a possible relation between GmRR34 and ABA biosynthesis (Figure 1A and Figure 5D). Meanwhile, OST1 had been documented by numerous studies and recognized as a core component of ABA signaling. In this pathway, its functions are associated with the regulation of stomatal closure by phosphorylating various membrane proteins and ion channels such as plasma membrane intrinsic proteins (PIPs), slow anion channel-associated 1 (SLAC1) and potassium channel in *Arabidopsis thaliana* 1 (KAT1) [79,84,85,86]. The other two downstream components of the ABA-signaling pathway, *ABI5* and *RAB18*, are ABA-induced genes and have been demonstrated to function in various abiotic stresses including water deprivation [87,88]. ABI5 is a basic leucine zipper TF, playing key roles in abiotic stress response through regulating gene expression of various downstream targets [88], whereas, RAB18 is a functional protein (i.e., dehydrin), which is responsible for cellular protection under water deficit conditions [87,89]. Furthermore, a previous study demonstrated that *CAT1* could be the downstream gene regulated by ABI5 [90], whereby their expression upon drought treatment were both up-regulated (Figure 3D and Figure 5D). Taken these results with lower water loss rates (importantly within the first h of dehydration) observed in the transgenic *Arabidopsis* together, it is suggested that GmRR34 enhances drought resistance of the transgenic plants in an ABA-dependent manner, at least by promoting the closure of stomata under water-deficit condition.

## 4. Materials and Methods

### 4.1. Plant Materials

WT *Arabidopsis* (Col-0) were used as control plants and materials for generating transgenic plants. *GmRR34* (Glyma03g32720) cDNA [35] was inserted into plasmid pBI121 by replacing the selective marker gene *β-glucuronidase* (*GUS*) and under the regulation of constitutive *Cauliflower mosaic virus* (*CaMV*) *35S* promoter. The recombinant vector was transferred into *Agrobacterium tumefaciens* strain EHA101 for generating transgenic plants by *Agrobacterium*-mediated floral dip method [91]. The transgenic plants were then screened for independent homozygous progenies based on the ratio of kanamycin-resistant over kanamycin-sensitive phenotypes (applied antibiotic at concentration of 30 mg L^−1^) over three successive generations from T1 (with 7 independent lines) to T3 according to the Mendelian genetic laws [92]. 

### 4.2. Growth Conditions

The methods for seed sterilization, germination and normal growth conditions were described in Nguyen et al. (2019) [42]. In brief, the seeds of studied lines were sterilized by ethanol and sodium hypochlorite prior to germination on Murashige and Skoog (MS) medium (1% sucrose, 0.8% agar). Treated seeds were stratified in the dark for 2 days before proceeding to normal growth condition using controlled facility (22 °C, 60% humidity and 16/8 light-dark cycle). In all experiments, except ABA sensitivity assay, the seeds were germinated and grown on MS medium for ten days. These seedlings were then transferred to soil (Tribat, Saigon Xanh Biotechnology Ltd. Company, Ho Chi Minh City, Vietnam), and allowed to grow normally in the same growth room for additional fourteen days before being subjected to the assays. Survival test and water-loss assay were performed separately. For the analysis of hydrogen peroxide content, enzyme activities, and expression of drought-related genes, the same set of plants were used.

### 4.3. Phenotypic Analyses of Ectopic Expression Lines under Normal Conditions

Root length of 24-day-old plants was measured by taking the plants out of soil without damaging the root system [42]. Length from the longest leaf of each plant was used to determine the maximum rosette radius, using the Image-J software (https://imagej.nih.gov/ij/) [41]. *Arabidopsis* rosette area measurement was carried out following the method described in a previous study [93] using Photoshop CC 2019 (Adobe, San José, CA, USA). Each experiment was performed with ten biological replicates.

### 4.4. Survival Test

The plants growing in tray system were subjected to drought stress by withholding water until the relative soil moisture content (SMC) dropped below 20%, using moisture meter (TK-100G, Yieryi, Guangdong, China) to measure randomly 6 different soil positions for each treatment per timepoint. Other growing factors were maintained the same as specified in Section 4.2. Following the drought treatment, the treated plants were re-irrigated for 3 days before recording the survival ratio of each genotype, of which the criterion was the capacity of developing new green tissues [42]. The experiment was triplicated, with 20 plants per replicate.

### 4.5. Determination of Excised-Leaf Water Loss Rate 

Aerial part from individual 24-day-old-plants were excised for performing water loss assay. In brief, the tissues were dehydrated at room temperature condition (25 °C) and their FWs were recorded at 1-h-intervals over a period of 5 h. Reduction in FWs during the studied course was used to estimate the water loss rates from the samples [94]. Nine plants of similar phenotype were used in this experiment for each genotype.

### 4.6. Evaluation of H_2_O_2_ Content and ROS-Scavenging Enzymatic Activities

Leaf tissues from plants that had been subjected to 10-day drought treatment were used for analyses (0.2 g leaves per plant as a replicate, three biological replicates). Endogenous H_2_O_2_ content was determined following Patterson’s procedure [95] with minor modification by using 0.1% titanium(III) sulfate and 20% sulfuric acid in preparing reaction solution. Previously described methods were followed for quantification of soluble proteins [96], evaluation of SOD [97] and CAT [98] enzymatic activities. The enzymes were extracted in potassium phosphate buffer (1 M, pH 7.8) containing EDTA 0.1 M and 2% polyvinylpyrrolidone (M.W. 8000). Bovine serum albumin (Sigma, Saint Louis, MO, USA) was used to establish the standard curve for protein quantification.

### 4.7. Expression Analysis of Transgene and Drought Response-Related Genes

To analyze expression of drought-related genes by RT-qPCR, the shoot tissues without inflorescence (3 biological replicates) of both 10-day-drought-treated seedlings and of the control plants grown under normal conditions were collected. For checking transgene expression, well-watered samples of transgenic plants were used, along with WT as negative control. Total RNA isolation, cDNA synthesis and RT-qPCR were conducted following the methods previously described in Hoang et al. (2020) [40]. Primers used in this experiment were obtained from previous studies (Supplementary Table S1). Relative expression level was analyzed by 2^−Δ*Ct*^ method [99], and *Actin2* was used as the reference gene [100].

### 4.8. ABA Sensitivity Assays

Plants were cultivated on MS medium without ABA and with ABA at concentration of 0.3 or 0.5 µM under normal growth conditions. To figure out the germination rates, the seeds with emerged radicles within the examined population were counted after 2 days of stratification followed by 3 days of incubation. To determine the rates of successful cotyledon development, seedlings with green cotyledons were counted after 7 days of incubation. These procedures were performed using methods described in previous study [101]. Each experiment was triplicated, with 100 seeds per replicate. For evaluation of root elongation, seeds of WT and transgenic lines were germinated on MS medium and allowed to grow for 4 days. After that, the seedlings were transferred to MS plates, supplemented with different concentrations of ABA (0, 0.3, and 0.5 µM) and let grow vertically for 8 days prior to the measurement of root length [102]. Ten biological replicates were used for each genotype for determining of root growth capacity under ABA treatment.

### 4.9. Statistical Analysis

To identify statistically significant difference among genotypes under the same treatment, data were analyzed using one-way ANOVA (Tukey’s honestly significant difference—HSD test).

## 5. Conclusions

The combined results from this study have demonstrated that GmRR34 plays as a positive regulator in mediating plant response to water deficit condition in an ABA-dependent manner, suggestively at least in *Arabidopsis* and/or soybean plant systems. Its biological functions have been found to be involved in various processes, including water retention ability, antioxidant enzyme activities, and regulating a number of drought-related genes.

## Supplementary Materials

The following are available online at https://www.mdpi.com/2223-7747/9/4/494/s1, Figure S1: Root growth characteristics of ABA-treated plants; Supplementary Table S1: List of primers used in the RT-qPCR.
