# Supplementary material for: Heterologous Expression of a Soybean Gene RR34 Conferred Improved Drought Resistance of Transgenic Arabidopsis
Source: Plants (Basel). 2020 Apr 12;9(4):494. doi: 10.3390/plants9040494 (PMC7238260; doi:10.3390/plants9040494)
Supplement: Supplementary file 1 [file plants-09-00494-s001.zip › Supplementary_Plants_757627/Figure S1_Supplementary_Plants_757627.docx]

| 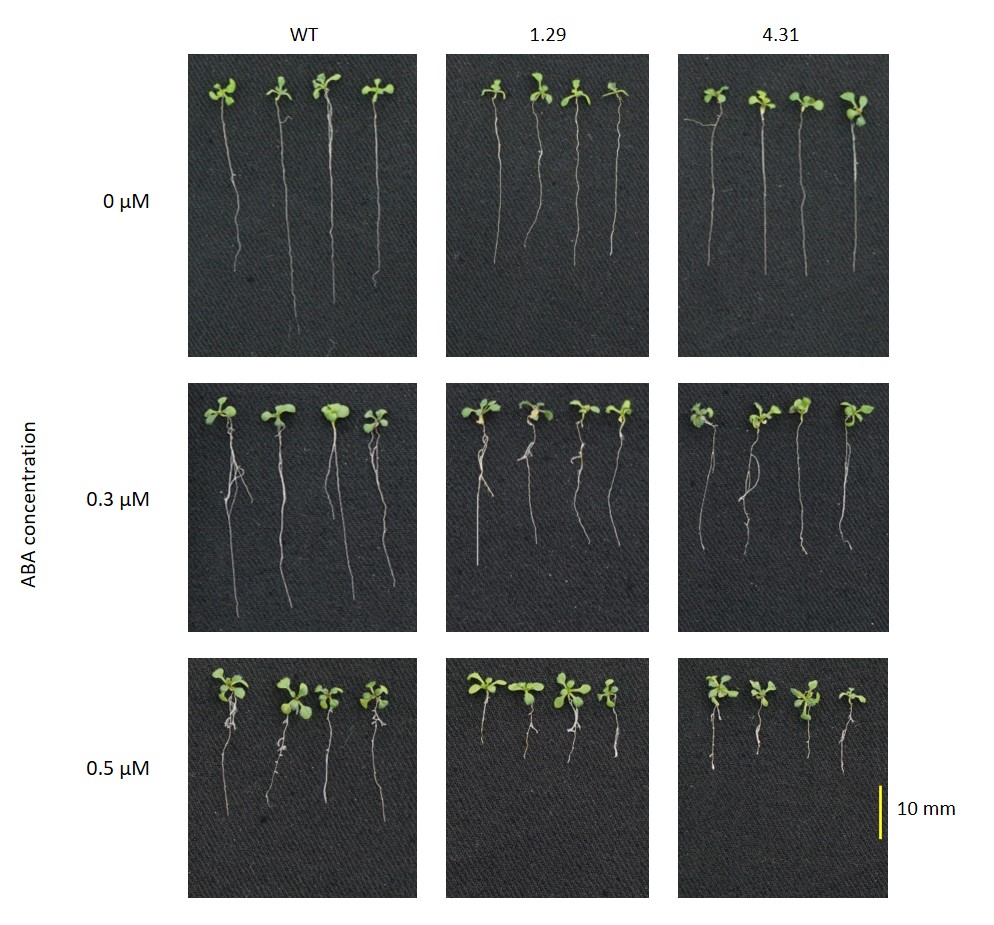 |
| --- |

**Figure S1.** Root growth characteristics of WT (wild-type) and *GmRR34*-carrying transgenic *Arabidopsis* (lines 1.29 and 4.31) grown on vertical MS plates, which were supplied with various concentrations of abscisic acid (ABA) (0, 0.3 and 0.5 µM of ABA) (n = 10).

| 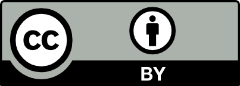 | © 2020 by the authors. Submitted for possible open access publication under the terms and conditions of the Creative Commons Attribution (CC BY) license (http://creativecommons.org/licenses/by/4.0/). |
| --- | --- |
