# Supplementary material for: Heterologous Expression of a Soybean Gene RR34 Conferred Improved Drought Resistance of Transgenic Arabidopsis
Source: Plants (Basel). 2020 Apr 12;9(4):494. doi: 10.3390/plants9040494 (PMC7238260; doi:10.3390/plants9040494)
Supplement: Supplementary file 1 [file plants-09-00494-s001.zip › Supplementary_Plants_757627/Table S1_Supplementary_Plants_757627.docx]

**Table S1.** List of primers used in the RT-qPCR.

| **Genes** | **ID** | **Forward (5’-3’)** | **Reverse (5’-3’)** | **References** |
| --- | --- | --- | --- | --- |
| *GmRR34* | Glyma03g32720 | GAGGCAACAAAGGAACTTCG | TCCGTACAGCGTGATGATACA | [1] |
| *AtActin2* | At3g18780 | GCACCACCTGAAAGGAAGTACA | CGATTCCTGGACCTGCCTCATC | [2] |
| *AtCAT1* | At1g20630 | TGGGATTCAGACAGGCAAGAACG | GTTTGGCCTCACGTTAAGACGAGT | [3] |
| *AtCSD1* | At1g08830 | AGACCCTGATGACCTCGGAAA | GCCACACACCAGAAGATACAC | [4] |
| *AtNCED3* | At3g14440 | CGGTGGTTTACGACAAGAACAA | CAGAAGCAATCTGGAGCATCAA | [3] |
| *AtABI5* | At2g36270 | AACGGGAGATTGCGGACATT | ACAGGGAACACTAGTAAAGCAGA | [5] |
| *AtOST1/SnRK2.6* | At4g33950 | CCACAGGTCCTTAAGACATCCC | GCATGACAGTAACTAACTCCTG | [6] |
| *AtRAB18* | At5g66400 | GCATAGACTTTGCTCGGGAGT | CCGCCAGACGAACCTTCA | [7] |
| *AtRD29A* | At5g52310 | TGGATCTGAAGAACGAATCTGATATC | GGTCTTCCCTTCGCCAGAA | [8] |
| *AtLEA14* | At1g01470 | AAGGACTTCGTGGCGGATAAA | CGGTTCACGTCTTTGAGGTCA | [9] |
| *AtHSP70B* | At1g16030 | GCAGAAGATTGAGAAGGCGATTG | CGCTCTAATCCACCTCTTCGATC | [10] |

References

1. Le, D.T.; Nishiyama, R.I.E.; Watanabe, Y.; Mochida, K.; Yamaguchi-Shinozaki, K.; Shinozaki, K.; Tran, L.-S.P. Genome-wide expression profiling of soybean two-component system genes in soybean root and shoot tissues under dehydration stress. *DNA Res.* **2011**, *18*, 17-29.

2. Yang, L.; Liu, Q.; Liu, Z.; Yang, H.; Wang, J.; Li, X.; Yang, Y. *Arabidopsis* C3HC4‐RING finger E3 ubiquitin ligase AtAIRP4 positively regulates stress‐responsive abscisic acid signaling. *J. Integr. Plant Biol.* **2016**, *58*, 67-80.

3. Nguyen, K.H.; Mostofa, M.G.; Li, W.; Van Ha, C.; Watanabe, Y.; Le, D.T.; Thao, N.P.; Tran, L.-S.P. The soybean transcription factor GmNAC085 enhances drought tolerance in *Arabidopsis*. *Environ. Exp. Bot.* **2018**, *151*, 12-20.

4. Chen, Y.; Jiang, J.; Song, A.; Chen, S.; Shan, H.; Luo, H.; Gu, C.; Sun, J.; Zhu, L.; Fang, W. Ambient temperature enhanced freezing tolerance of *Chrysanthemum dichrum* *CdICE1* *Arabidopsis* via miR398. *BMC Biol.* **2013**, *11*, 121.

5. Huang, Q.; Wang, Y.; Li, B.; Chang, J.; Chen, M.; Li, K.; Yang, G.; He, G. TaNAC29, a NAC transcription factor from wheat, enhances salt and drought tolerance in transgenic *Arabidopsis*. *BMC Plant Biol.* **2015**, *15*, 268.

6. Ding, Y.; Li, H.; Zhang, X.; Xie, Q.; Gong, Z.; Yang, S. OST1 kinase modulates freezing tolerance by enhancing ICE1 stability in *Arabidopsis*. *Dev. Cell* **2015**, *32*, 278-289.

7. Hao, Y.J.; Wei, W.; Song, Q.X.; Chen, H.W.; Zhang, Y.Q.; Wang, F.; Zou, H.F.; Lei, G.; Tian, A.G.; Zhang, W.K. Soybean NAC transcription factors promote abiotic stress tolerance and lateral root formation in transgenic plants. *Plant J.* **2011**, *68*, 302-313.

8. Rasheed, S.; Bashir, K.; Matsui, A.; Tanaka, M.; Seki, M. Transcriptomic analysis of soil-grown *Arabidopsis* *thaliana* roots and shoots in response to a drought stress. *Front. Plant Sci.* **2016**, *7*, 180.

9. Hundertmark, M.; Hincha, D.K. LEA (late embryogenesis abundant) proteins and their encoding genes in *Arabidopsis* *thaliana*. *BMC Genomics* **2008**, *9*, 118.

10. Echevarría‐Zomeño, S.; Fernández‐Calvino, L.; Castro‐Sanz, A.B.; López, J.A.; Vázquez, J.; Castellano, M.M. Dissecting the proteome dynamics of the early heat stress response leading to plant survival or death in *Arabidopsis*. *Plant Cell Environ.* **2016**, *39*, 1264-1278.

| 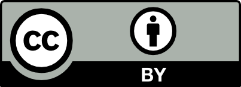 | © 2020 by the authors. Submitted for possible open access publication under the terms and conditions of the Creative Commons Attribution (CC BY) license (http://creativecommons.org/licenses/by/4.0/). |
| --- | --- |
